# The post-COVID-19 population has a high prevalence of cross-reactive antibodies to spikes from all *Orthocoronavirinae* genera

**DOI:** 10.1128/mbio.02250-23

**Published:** 2023-12-19

**Authors:** Gagandeep Singh, Anass Abbad, Giulio Kleiner, Komal Srivastava, Charles Gleason, PARIS Study Group, Dalles Andre, Juan Manuel Carreño, Viviana Simon, Florian Krammer

**Affiliations:** 1 Department of Microbiology, Icahn School of Medicine at Mount Sinai, New York, New York, USA; 2 Center for Vaccine Research and Pandemic Preparedness (C-VaRPP), Icahn School of Medicine at Mount Sinai, New York, New York, USA; 3 Department of Pathology, Molecular and Cell-Based Medicine, Icahn School of Medicine at Mount Sinai, New York, New York, USA; 4 Division of Infectious Diseases, Department of Medicine, Icahn School of Medicine at Mount Sinai, New York, New York, USA; 5 The Global Health and Emerging Pathogens Institute, Icahn School of Medicine at Mount Sinai, New York, New York, USA; Johns Hopkins University, Baltimore, Maryland, USA

**Keywords:** SARS-CoV-2 immunity, antibodies, cross-reactivity, Orthocoronavirinae

## Abstract

The Orthocoronavirinae subfamily is large comprising four highly divergent genera. Four seasonal coronaviruses were circulating in humans prior to the coronavirus disease 2019 pandemic. Infection with these viruses induced antibody responses that are relatively narrow with little cross-reactivity to spike proteins of other coronaviruses. Here, we report that infection with and vaccination against severe acute respiratory syndrome coronavirus 2 (SARS-CoV-2) induces broadly cross-reactive binding antibodies to spikes from a wide range of coronaviruses including members of the sarbecovirus subgenus, other betacoronaviruses including Middle Eastern respiratory syndrome coronavirus, and extending to alpha-, gamma-, and delta-coronavirus spikes. These data show that the coronavirus spike antibody landscape in humans has profoundly been changed and broadened as a result of the SARS-CoV-2 pandemic. While we do not understand the functionality of these cross-reactive antibodies and their impact, there is the possibility that they may lead to enhanced resistance of the population to infection with newly emerging coronaviruses with pandemic potential.

**IMPORTANCE:**

As demonstrated by severe acute respiratory syndrome coronavirus 2, coronaviruses pose a significant pandemic threat. Here, we show that coronavirus disease 2019 mRNA vaccination can induce significant levels of cross-reactive antibodies against diverse coronavirus spike proteins. While these antibodies are binding antibodies that likely have little neutralization capacity and while their contribution to cross-protection is unclear, it is possible that they may play a role in protection from progression to severe disease with novel coronaviruses.

## INTRODUCTION

Severe acute respiratory syndrome coronavirus 2 (SARS-CoV-2) infections and vaccinations induce binding and neutralizing antibodies to the spike protein of this new virus in humans (1, 2). Initially, these responses led to protection from symptomatic disease as shown in a number of clinical trials (3, 4). However, with the emergence of variants of concern, especially Omicron and its sub-variants, protection against symptomatic disease decreased since these new variants escaped the neutralizing antibody response induced by spike proteins from the ancestral SARS-CoV-2. However, it has been reported that binding antibodies to spike protein are much better maintained against the variants as compared to neutralizing activity (5). These binding antibodies may—in addition to T-cell immunity—contribute to the mostly maintained protection from severe disease (6) as has been shown by a recent study (7).

## RESULTS

### COVID-19 mRNA vaccines induce antibodies to diverse coronavirus spike proteins

**Fig 1:**
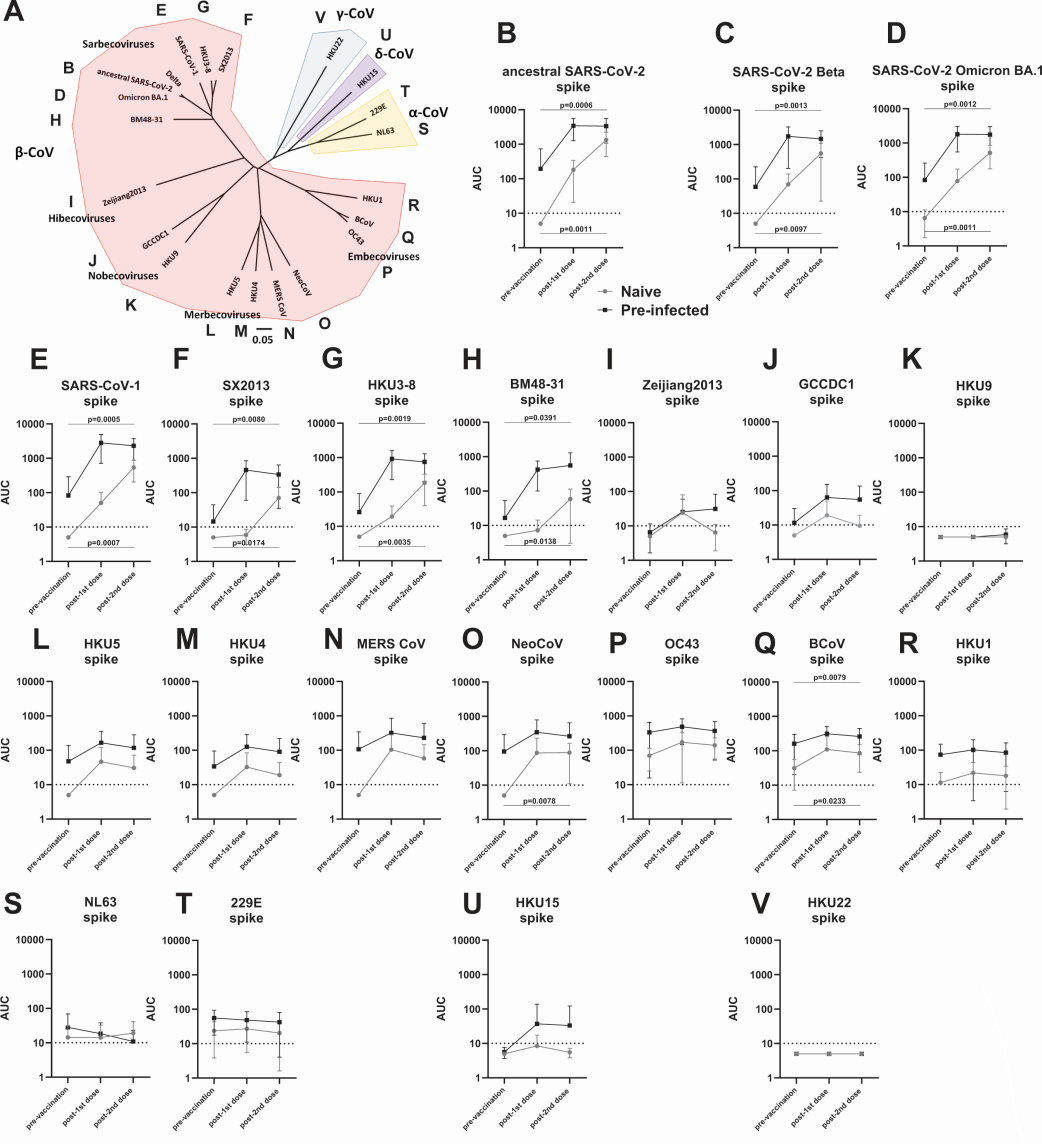
Induction of antibodies to diverse *Orthocoronavirinae* spike proteins. (A) A phylogenetic tree built with amino acid sequences of the spike proteins used in this study. The tree was built using Clustal Omega, visualized in FigTree and labels, and highlighting was added in Microsoft Powerpoint. The scale bar indicates a 5% change in amino acid sequence. Accession numbers and full name of the different virus strains used can be found in Table S1. (B–D) Reactivity of longitudinal serum samples from naïve (black) or SARS-CoV-2 pre-infected (gray) individuals at a pre-vaccination time point, after the first dose of COVID-19 mRNA vaccine and after the second dose of COVID-19 mRNA against the ancestral spike of SARS-CoV-2, against the Beta variant spike and against the Omicron BA.1 variant spike. (C–R) Reactivity to diverse β-CoV spikes, (S–T) reactivity to α-CoV spikes, (U) reactivity to a δ-CoV spike, and (V) reactivity to a γ-CoV spike. *N* = 10 per group. Subject characteristics can be found in Table S2. Mean and standard deviation are shown in (B–V). For statistical analysis, a paired *t*-test between pre-vaccination and post-2nd vaccination titers was performed separately for naïve and pre-infected individuals. *P* values are indicated if they reached the arbitrary threshold of *P* = 0.05.

Here, we wanted to explore how cross-reactive antibodies induced by SARS-CoV-2 infection or vaccination bind beyond the spike protein of SARS-CoV-2 and its variants. We expressed a panel of coronavirus spikes representative of all *Orthocoronavirinae* genera. We generated 21 recombinant spikes representing the five beta-coronavirus (β-CoV) subgenera (sarbecoviruses, hibecoviruses, merbecoviruses, nobecoviruses, and embecoviruses) as well the alpha-coronavirus (α-CoV), gamma-coronavirus (γ-CoV), and delta-coronavirus (δ-CoV) genera (Fig. 1A; Table S1). Of note, for a number of these spikes, antigenicity and immunogenicity have not been determined. Using an established enzyme-linked immunosorbent assay (ELISA) (8), we tested longitudinal sera from 10 individuals who received the mRNA coronavirus disease 2019 (COVID-19) vaccine and from 10 individuals who received the vaccine after an initial SARS-CoV-2 infection (Table S2). Sera were taken before vaccination, post-1st dose (range 16–25 days for the vaccine-only group and 15–23 days for the infection-vaccination group), and post-2nd dose (range 14–28 days for the vaccine-only group and 16–29 days for the infection-vaccination group). Binding to SARS-CoV-2 ancestral and variant spikes was induced by vaccination as expected and was detectable before vaccination in people with pre-existing immunity (Fig. 1B through D). Comparable binding was found for the SARS-CoV-1 spike, and titers were also high against three other non-SARS-CoV-2 sarbecovirus spikes tested (Fig. 1E through H). In addition, an increase in binding was detected to a hibecovirus spike and merbecovirus spikes although at a lower level (Fig. 1I and L through O). COVID-19 vaccine-associated increases were only detected for one of the two nobecovirus spikes tested (Fig. 1J and K) but reactivity to embecoviruses was induced to some degree (Fig. 1P through R) and, as expected, higher at pre-vaccination baseline since two of the spikes tested are from embecoviruses circulating in humans (OC43 and HKU1). No increase in reactivity was detected against the seasonal α-CoV spikes but pre-vaccination baseline titers to 229E spike were detectable while reactivity to NL63 spike was much lower (Fig. 1S and T). Interestingly, there was also an induction of antibodies to the γ-CoV spike of HKU15 but not to the δ-CoV spike of HKU22 (Fig. 1U and V). In general, most SARS-CoV-2 infected individuals already had titers to these spikes even before they got vaccinated. These data suggested that SARS-CoV-2 infection and vaccination can induce cross-reactive anti-spike antibodies. Of note, the sample size for this analysis was not determined using a power calculation since it is exploratory work and effect sizes were unknown. Statistical tests were performed and in several cases, increases in antibody titers were significant. However, the study was not designed to determine statistically significant differences.

### The post-COVID-19 spike cross-reactivity landscape is dramatically different from the pre-COVID-19 spike cross-reactivity landscape

**Fig 2:**
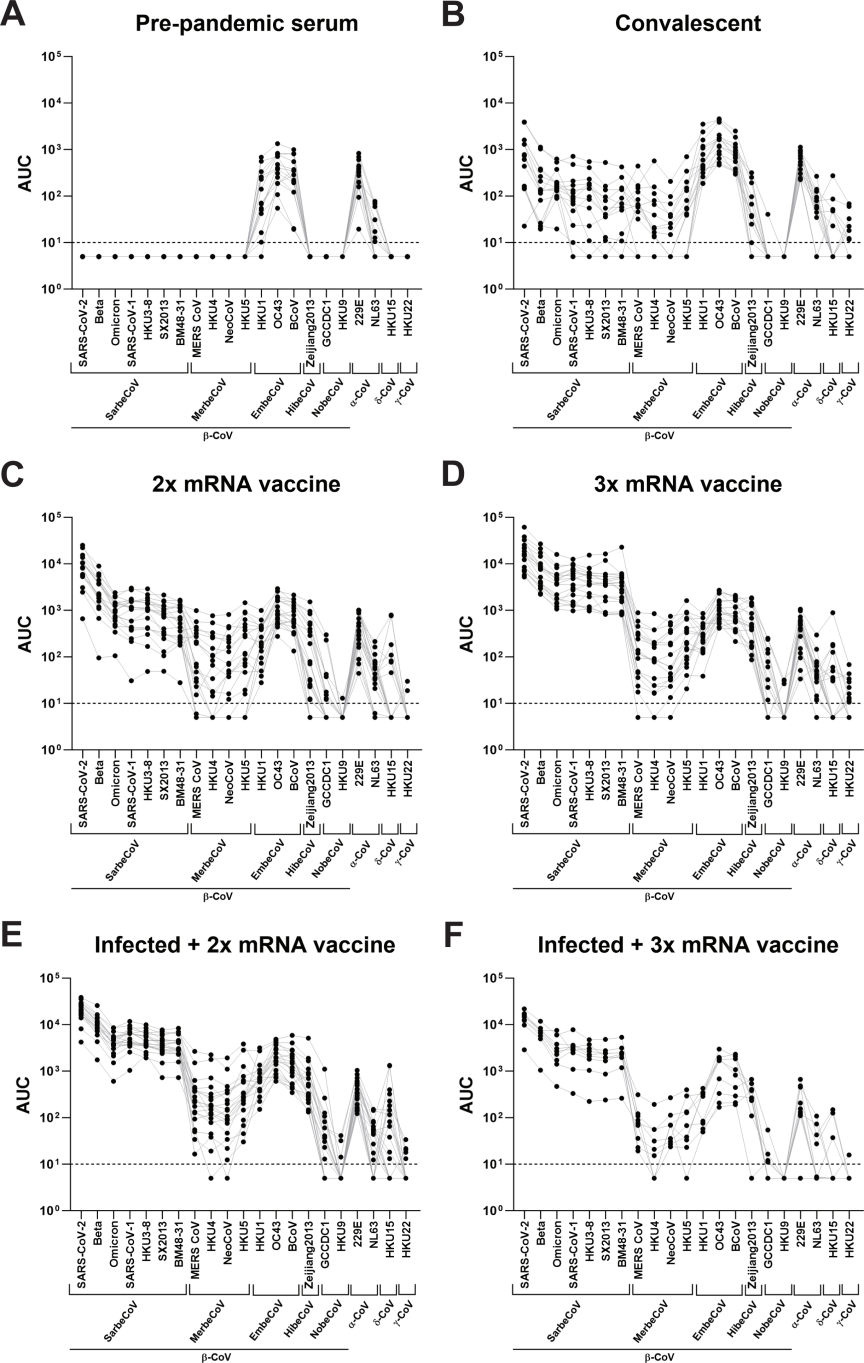
Cross-sectional reactivity of human sera from different exposure groups to diverse *Orthocoronavirinae* spike proteins. (A) Serum reactivity of pre-pandemic serum samples to different spike proteins (*n* = 15). (B) Serum reactivity of SARS-CoV-2 convalescent individuals to different spike proteins (*n* = 15). (C) Serum reactivity of individuals vaccinated 2x with COVID-19 mRNA vaccines (n=20). (D) Serum reactivity of individuals who got three doses of vaccine (*n* = 19) to different spike proteins. (E and F) Serum reactivity of individuals infected with SARS-CoV-2 and then vaccinated twice (*n* = 20 except for SX2013 where *n* = 19) or three times (*n* = 9) to different spike proteins. Subject characteristics can be found in Table S3.

**Fig 3:**
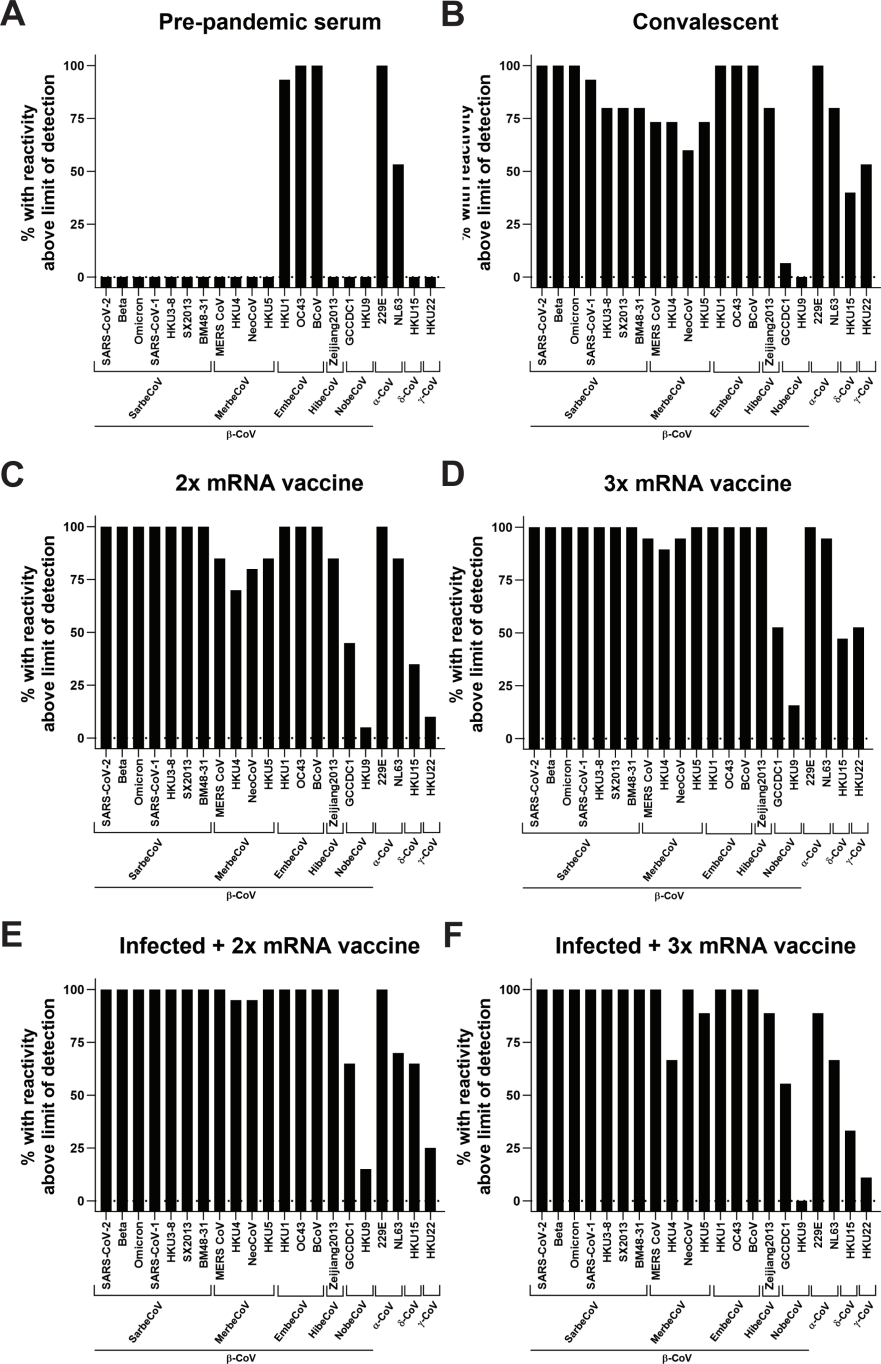
Percentage of individuals with reactivity above the LoD to different spike proteins for each group shown in Fig. 1. (A) Percentage of pre-pandemic serum samples reactive to different spike proteins (*n* = 15). (B) Percentage of samples from SARS-CoV-2 convalescent individuals reactive to different spike proteins (*n* = 15). (C) Percentage of individuals vaccinated 2x with COVID-19 mRNA vaccines reactive to different spikes (n=20) and (D) the same for individuals who got three doses of vaccine (*n* = 19). (E and F) Percentage of individuals infected with SARS-CoV-2 and then vaccinated twice (*n* = 20 except for SX2013 where *n* = 19) or three times (*n* = 9) who had reactivity to different spike proteins. Subject characteristics can be found in Table S3.

We next investigated how different immune histories influence pan-coronavirus seroreactivity. We tested sera from different exposure groups including pre-pandemic samples (*n* = 15, collected between 2018 and 2019), samples from convalescent individuals (*n* = 15, collected at 23–87 days post-infection), individuals who got two doses of COVID-19 mRNA vaccines (*n* = 20, range of 14–36 days post-2nd dose), individuals who got three doses of COVID-19 mRNA vaccines (*n* = 19, range of 14–33 days post-3rd dose), individuals who were infected and then got two doses of COVID-19 mRNA vaccines (*n* = 20, range of 15–39 days post-2nd dose), and individuals who got infected and three doses of COVID-19 mRNA vaccines (*n* = 9, range of 14–30 days post-3rd dose). More information regarding the specific samples can be found in Table S3. Analysis of pre-pandemic sera showed reactivity to the β-CoV spikes from two human seasonal coronaviruses, OC43 and HKU1. Interestingly, there was also strong reactivity, comparable to OC43, to the spike of the bovine coronavirus (BCoV) with sera from all study participants having reactivity above the limit of detection (LoD) (Fig. 2A and 3A). Reactivity against spikes from seasonal α-CoVs, NL63, and 229E was also found. Similar to data shown in Fig. 1S and T, reactivity to NL63 was much lower than for 229E. For sera from SARS-CoV-2 convalescent individuals, we observed strong reactivity to SARS-CoV-2 spikes with elevated reactivity to spikes of many β-CoV with the exception of the nobecovirus spikes from GCCDC1 and HKU9 (Fig. 2B). Sera from several study participants (40% and 53.3%, respectively) even had detectable reactivity to the γ-CoV spike of HKU15 and δ-CoV spike of HKU22 (Fig. 3B). By receiving two or three doses of COVID-19 mRNA vaccines, the reactivity pattern changed slightly. With two doses of the vaccine, higher reactivity to β-CoV spikes could be observed (Fig. 2C). With three doses of the mRNA vaccine, high and relatively uniform reactivity to sarbecoviruses could be measured (Fig. 2D). In addition, the percentage of individuals who had detectable reactivity in the 2× and 3× mRNA vaccinated groups was high across all tested spikes (Fig. 3C and D). Sera from people who had received three vaccine doses cross-reacted with all spikes except the spikes of the nobecoviruses GCCDC1 and HKU9 and the γ-CoV spike of HKU15 and to the δ-CoV of HKU22 where approximately 15.8%–52.6% of individuals had reactivity (Fig. 3D). Similarly, strong reactivity across the board was also detected in individuals who had been infected and then vaccinated 2× or 3× with COVID-19 mRNA vaccines (Fig. 2E and F, Fig. 3E and F).

## DISCUSSION

Several of our observations from these experiments are interesting. Even in pre-COVID-19 sera, the prevalence and titers against the spike of β-CoV BCoV are almost as high as against the spike of seasonal β-CoV OC43. This indicates an antigenic relatedness of the BCoV and OC43 spikes. It has been hypothesized that OC43 shares a common ancestor with BCoV and may have split off around 1890 (9). This split would coincide with the “Russian flu” pandemic of 1889/1890, and it has been hypothesized that this pandemic was, indeed, caused by an OC43 ancestor that jumped from cattle into humans (10). The cross-reactivity found may perhaps further support this hypothesis and also explain why zoonotic infections in humans with BCoV are rare even though the virus can infect children (11). In addition, the titers as well as the percentage of positive individuals for the seasonal α-CoV 229E are much higher than for the seasonal α-CoV NL63. These differences may point perhaps to an inherent difference in immunogenicity and antibody durability or represent an artifact of the protein used in the ELISA. Furthermore, from our longitudinal sample set, we noted that COVID-19 mRNA vaccination induces antibodies to diverse spike proteins including many β-CoVs spikes as well as δ-CoV spikes. As previously reported, backboosting to OC43 and HKU (and BCoV) was also observed (12, 13), but little to no induction of antibodies was seen against α-CoV spikes, the tested γ-CoV spike, or the spikes from the nobecoviruses subgenus of the β-CoV. At a cross-sectional level, we observed that sera from individuals infected with SARS-CoV-2 already show relatively broad reactivity across β-CoV spikes with about half of the individuals having also some reactivity to δ-CoV and γ-CoV spikes. COVID-19 mRNA vaccination induced similar cross-reactivity. However, especially after three vaccine doses, there seems to be a bias toward sarbecoviruses with a clear drop toward other non-seasonal β-CoV spikes. It is important to note that after mRNA vaccination with the original vaccine containing the ancestral SARS-CoV-2 spike, titers against diverse sarbecovirus spikes are as high as against the Omicron spike. This may suggest, that the protection from severe disease that we observe against Omicron sublineages perhaps also applies to other, diverse sarbecoviruses (e.g., SARS-CoV-1, HKU3-8, SX2013, BM48-31) which may (re)-emerge in the future as human pathogens. While these cross-reactive antibodies may not neutralize more distantly related viruses, they may still afford protection via Fc-mediated effector functions. Binding antibodies have been correlated with protection by Earle and colleagues early in the pandemic (14). Importantly, a recent study has shown that in the absence of robust neutralizing antibodies, binding antibodies are associated with protection from progression to severe COVID-19 and death (7). Nevertheless, at this point in time, it remains unclear if these cross-reactive binding antibodies do in fact provide a clinical benefit. Of note, vaccine-enhanced disease has been observed in some animal models for SARS-CoV-1 and Middle Eastern respiratory syndrome coronavirus (MERS CoV) (while in other models protection was found). It is unclear if these observations are related to antibodies or not. However, this disease enhancement has not been observed for SARS-CoV-2 or for any coronavirus in humans. Actually, as mentioned above, non-neutralizing binding antibody has been connected to protection (7). We therefore do not think that these cross-reactive antibodies do increase risk.

Another interesting aspect is the question of which epitopes these cross-reactive antibodies target. Cross-reactivity within the sarbecoviruses likely targets the receptor binding domain (RBD) but also different epitopes on the S2 subunit. Cross-reactivity to other β-CoV spikes is likely mediated mostly by S2 targeting antibodies since the RBD is probably too divergent. Such antibodies have been isolated with some of them having neutralizing activity although at lower potency (often μg/mL range) than RBD targeting mAbs which can, in some cases, reach the ng/mL range (12, 15–18). Finally, cross-reactivity to α-CoV, δ-CoVs, and γ-CoVs is likely due to more rare antibodies that target the fusion peptide in the S2 domain. These antibodies have also been isolated and they exert neutralizing activity to some extent (19, 20). However, it would be helpful to isolate more mAbs with different reactivity profiles to understand their characteristics better and determine if they indeed can contribute to protection *in vivo*. Of note, monoclonal antibody therapeutics and prophylactics have been very efficient treatment options for COVID-19, but antigenic changes of SARS-CoV-2 have rendered them irrelevant. Perhaps antibodies that target more conserved epitopes, even if they have lower neutralizing potency, could be a more sustainable solution.

In summary, we found a high prevalence of antibodies that cross-react to spike proteins from all four *Orthocoronavirinae* genera. It is entirely possible that the global population, which is hyperimmunized to SARS-CoV-2 through infection and vaccination, has now built more resistance to the many members of the coronavirus family in general.

## MATERIALS AND METHODS

### Recombinant proteins expression

Mammalian expression vectors encoding the ectodomain of spike proteins from SARS-CoV-1 (GenBank: AAP13441.1), SARS-CoV-2 (GenBank: MN908947.3), HKU3-8 (GenBank: ADE34766.1), SX2013 (GenBank: AIA62300.1), BM48-31(GenBank: YP_003858584.1), SARS-CoV-2 Omicron (GenBank: UFT26501.1), MERS-CoV (GenBank: AXP07355.1), HKU4 (GenBank: YP_001039953.1), HKU5 (GenBank: YP_001039962.1), HKU9 (GenBank: YP_001039971.1), GCCDC1 (GenBank: QKF94914.1), Zhejiang2013, bat Hp (GenBank: YP_009072440.1) HKU15 (GenBank: YP_009513021.1), HKU22 (GenBank: AHB63508.1), BCoV (GenBank: AAA66399.1), Neo CoV, Coronavirus Neoromicia (GenBank: AGY29650.2), 229E (GenBank: NP_073551.1), NL63 (GenBank: AFV53148.1), OC43 (GenBank: KF963240.1), and HKU1 (GenBank: AGW27881.1) (see Table S1 for more information on virus isolates, host, and receptor) with a C-terminal thrombin cleavage site, T4 foldon trimerization domain and hexahistidine tag were constructed as described earlier (8, 21). The constructs also contain the “2P” stabilizing mutations (22) and known or putative S1-S2 cleavage sites were removed. Proteins were purified from transiently transfected Expi293F cells with each respective plasmid. Cell-free supernatant was harvested after 3 days post-transfection, and his-tagged proteins were purified by gravity flow chromatography using Ni^2+^-nitrilotriacetic acid agarose (Qiagen). Proteins were eluted, buffer was exchanged using Amicon centrifugal units (EMD Millipore), and all recombinant proteins were finally re-suspended in phosphate buffered saline (PBS) as described (21). Proteins were run on sodium dodecyl sulfate-polyacrylamide gel electrophoresis gels under reducing conditions for quality control and stored at −80°C until use.

### ELISA

Antibody titers in in serum samples were assessed using a research grade ELISA (8, 21) using recombinant versions of full-length spike (S) proteins of different coronaviruses. Briefly, Immulon 4 HBX 96-well microtiter plates (Thermo Fisher Scientific) were coated overnight at 4°C with 50 µL per well of a 2 µg/mL solution of each respective recombinant protein resuspended in PBS (Gibco; cat. no. 10010-031). The next morning, plates were washed three times with PBS supplemented with 0.1% Tween-20 (PBS-T) using an automatic plate washer (BioTek 405TS microplate washer) and blocked with 200 µL per well of PBS-T containing 3% milk powder (AmericanBio) for 1 h at room temperature (RT). Blocking solution was removed, and initial dilutions (1:100) of heat-inactivated serum (in PBS-T 1% milk powder) were added to the plates, followed by twofold serial dilutions and 2 h incubation at RT. Next, plates were washed three times with PBS-T, and 50 µL per well of the pre-diluted secondary anti-human IgG (Fab-specific) horseradish peroxidase antibody (produced in goat; Sigma-Aldrich, cat. no. A0293, RRID: AB_257875) diluted 1:9,000 in PBS-T containing 1% milk powder was added for 1 h. Plates were again washed three times with 0.1% PBS-T and the substrate o-phenylenediamine dihydrochloride (SIGMAFAST) was added (100 µL per well) for 10 min, followed by an addition of 3 M hydrochloric acid (50 µL per well; Thermo Fisher Scientific) to stop the reaction. Optical density was measured at a wavelength of 490 nm using a plate reader (BioTek, Synergy H1 microplate reader). The area under the curve values were calculated and plotted using Prism 9 software (GraphPad).

### Human serum samples

Human serum samples were obtained from study participants in the longitudinal observational Protection Associated with Rapid Immunity to SARS-CoV-2 (PARIS) study (23). This cohort follows healthcare workers longitudinally since April 2020. The study was reviewed and approved by the Icahn School of Medicine at Mount Sinai Institutional Review Board (IRB-20-03374). All participants provided informed consent and HIPAA Authorization prior to sample and data collection. All participants provided permission for sample banking and sharing. The participants did not receive compensation. All biospecimens were coded and stored at −80°C.

We used longitudinal serum samples collected from 20 adult study participants. Ten out of 20 of the study participants (50%) were infected with SARS-CoV-2 prior to the first vaccine dose and were seropositive prior to vaccination (pre-infected group). Ten out of 20 study participants (50%) had no previous SARS-CoV-2 infection history and were seronegative for SARS-CoV-2 antibodies prior to vaccination (naive group). The majority of the other 50% were infected in the first wave, with symptom onset at least 180 days prior to receiving the first dose of the mRNA vaccine, which was confirmed through antibody testing on samples collected prior to vaccination. A few participants experienced infection within 45 days of the first dose; these were confirmed by PCR testing. All infections were mild. Participants received two doses of either the Moderna mRNA-1273 vaccine or the Pfizer-BioNTech BNT162b2 vaccine. Demographics of seropositive and seronegative study participants and sample collection time points from each individual are summarized in Table S2.

For the antigenic landscape characterization against various coronaviruses, we selected 83 serum samples from 53 participants. Twenty out of 53 participants were seronegative prior to vaccination, while 33/53 had COVID-19 prior to vaccination. All participants with pre-vaccination immunity were infected in 2020 when only ancestral SARS-CoV-2 strains circulated in the New York metropolitan area. If testing was done, we used the date of the first positive molecular test as the date of infection. If no testing was available, the date of symptom onset was used for the timing of infection given that the participant also had a positive antibody test on a sample collected prior to vaccination. All infections were mild. Convalescent samples (*n*  =  15) were obtained within 3 months of SARS-CoV-2 infection (average: 58 days, range: 23–87 days), whereas the post-vaccination samples were collected on average 23 days (range: 14–39 days) after the second dose [*n*  =  40; where *n* = 20 Pfizer 2× (10 individuals with prior infection and 10 individuals with no infection) and *n* = 20 Moderna 2× (10 individuals with prior infection and 10 individuals with no infection)] or 19 days (range: 14–33 days) after the third booster [*n*  =  30; 18 Pfizer 3× (9 individuals with prior infection and 9 individuals with no infection) and 10 Moderna 3×] vaccine dose. Pre-pandemic human serum samples were collected before the COVID-19 pandemic. Demographics of participants and sample collection time points from each individual are summarized in Table S3.

## Data Availability

All data will be available from ImmPort under the following identifiers: SDY2412.

## SUPPLEMENTAL MATERIAL

Supplemental Material mbio.02250-23-s0001.docx Tables S1-S3.

ASM does not own the copyrights to Supplemental Material that may be linked to, or accessed through, an article. The authors have granted ASM a non-exclusive, world-wide license to publish the Supplemental Material files. Please contact the corresponding author directly for reuse.
