# Supplementary material for: The post-COVID-19 population has a high prevalence of cross-reactive antibodies to spikes from all Orthocoronavirinae genera
Source: mBio. 2023 Dec 19;15(1):e02250-23. doi: 10.1128/mbio.02250-23 (PMC10790767; doi:10.1128/mbio.02250-23)
Supplement: Supplemental Material — Tables S1-S3. [file mbio.02250-23-s0001.docx]

**Supplemental Material for:**

**The post-COVID-19 population has a high prevalence of crossreactive antibodies to spikes from all *Orthocoronavirinae* genera**

Gagandeep Singh^1,2^, Anass Abbad^1,2^, Giulio Kleiner^1,2^, Komal Srivastava^1,2^, Charles Gleason^1,2^, PARIS Study Group^1,2^, Juan Manuel Carreño^1,2^, Viviana Simon^1,2,3,4,5^ and Florian Krammer^1,2,3^*

*^1^Department of Microbiology, Icahn School of Medicine at Mount Sinai, New York, NY, USA*

*^2^Center for Vaccine Research and Pandemic Preparedness (C-VaRPP), Icahn School of Medicine at Mount Sinai, New York, NY, USA*

*^3^Department of Pathology, Molecular and Cell-Based Medicine, Icahn School of Medicine at Mount Sinai, New York, NY, USA*

*^4^ Division of Infectious Diseases, Department of Medicine, Icahn School of Medicine at Mount Sinai, New York, NY, USA*

*^5^The Global Health and Emerging Pathogens Institute, Icahn School of Medicine at Mount Sinai, New York, NY, USA*

*To whom correspondence should be addressed: [florian.krammer@mssm.edu](mailto:florian.krammer@mssm.edu)

**Supplementary Tables**

**Supplementary Table 1. Information on the viral isolates used for recombinant spike protein generation.**

| **Virus** | **Genus (subgenus)** | **Accession #** | **Host Species** | **Receptor** | **Reference** |
| --- | --- | --- | --- | --- | --- |
| 229E | α-CoV | NP_073551.1 | *Homo sapiens* | Human aminopeptidase N  (APN) | (1) |
| NL63 | α-CoV | AFV53148.1 | *Homo sapiens* | Angiotensin converting enzyme 2 (ACE2) | (2) |
| SARS CoV-2 | β-CoV  (sarbecovirus) | MN908947.3 | *Homo sapiens* | ACE2 | (3) |
| SARS-CoV-1 | β-CoV  (sarbecovirus) | AAP13441.1 | Several species | ACE2 | (4) |
| HKU3-8 | β-CoV  (sarbecovirus) | ADE34766.1 | *Rhinolophus* species |  | (5) |
| BM48-31 | β-CoV  (sarbecovirus) | YP_003858584.1 | *Rhinolophus blasii* |  | (5) |
| SX2013 | β-CoV  (sarbecovirus) | AIA62300.1 | *Rhinolophus ferrumequinum* |  | (5) |
| MERS CoV | β-CoV  (merbecovirus) | AXP07355.1 | Camelus dromedariu | Dipeptidyl peptidase 4 (DPP4) | (6) |
| HKU4 | β-CoV  (merbecovirus) | YP_001039953.1 | *Tylonycteris* species | DDP4 | (7, 8) |
| HKU5 | β-CoV  (merbecovirus) | YP_001039962.1 | *Pipistrellus* species |  | (7, 9) |
| NeoCoV | β-CoV  (merbecovirus) | AGY29650.2 | Neoromicia capensis | ACE2 | (10, 11) |
| HKU9 | β-CoV  (nobecovirus) | YP_001039971.1 | *Rousettus leschenaulti* |  | (9) |
| GCCDC1 | β-CoV  (nobecovirus) | QKF94914.1 | *Rousettus leschenaulti* |  | (12) |
| Zhejiang2013 (Bat Hp-betacoronavirus/Zhejiang2013) | β-CoV  (hibecovirus) | YP_009072440.1 | *Hipposideros pratti* |  | (13) |
| BCoV | β-CoV  (embecovirus) | AAA66399.1 | *Bovine* species | N-acetyl-9-O-acetylneuraminic acid (Neu5,9Ac2). | (14) |
| OC43 | β-CoV  (embecovirus) | KF963240.1 | *Homo sapiens sapiens* | N-acetyl-9-O-acetylneuraminic acid (Neu5,9Ac2). | (15) |
| HKU1 | β-CoV  (embecovirus) | AGW27881.1 | *Homo sapiens sapiens* | N-acetyl-9-O-acetylneuraminic acid (Neu5,9Ac2). | (15) |
| HKU15 | δ-CoV | YP_009513021.1 | *Sus scrofus* | porcine aminopeptidase N (pAPN) | (16) |
| HKU22 | γ-CoV | AHB63508.1 | *Tursiops* species |  | (17) |

**Supplementary Table 2: Characteristics of individuals shown in Figure 1.**

| **Participant ID** | **Age Bracket** | **Sex** | **Time points included in this study** | **SARS CoV-2 infection prior to vaccination** | **Vaccine type** |
| --- | --- | --- | --- | --- | --- |
| Pluto-001 | 45-49 | Male | Pre-vaccine, Post-1^st^ dose, Post-2^nd^ dose | Yes | Pfizer |
| Pluto-002 | 20-24 | Female | Pre-vaccine, Post-1^st^ dose, Post-2^nd^ dose | Yes | Moderna |
| Pluto-003 | 55-59 | Female | Pre-vaccine, Post-1^st^ dose, Post-2^nd^ dose | No | Moderna |
| Pluto-004 | 50-54 | Female | Pre-vaccine, Post-1^st^ dose, Post-2^nd^ dose | Yes | Pfizer |
| Pluto-005 | 40-44 | Female | Pre-vaccine, Post-1^st^ dose, Post-2^nd^ dose | No | Moderna |
| Pluto-006 | 35-39 | Female | Pre-vaccine, Post-1^st^ dose, Post-2^nd^ dose | No | Pfizer |
| Pluto-007 | 65-69 | Male | Pre-vaccine, Post-1^st^ dose, Post-2^nd^ dose | No | Moderna |
| Pluto-008 | 50-54 | Female | Pre-vaccine, Post-1^st^ dose, Post-2^nd^ dose | Yes | Pfizer |
| Pluto-009 | 25-29 | Female | Pre-vaccine, Post-1^st^ dose, Post-2^nd^ dose | Yes | Pfizer |
| Pluto-0010 | 25-29 | Female | Pre-vaccine, Post-1^st^ dose, Post-2^nd^ dose | No | Moderna |
| Pluto-0011 | 60-64 | Male | Pre-vaccine, Post-1^st^ dose, Post-2^nd^ dose | Yes | Pfizer |
| Pluto-0012 | 25-29 | Male | Pre-vaccine, Post-1^st^ dose, Post-2^nd^ dose | No | Moderna |
| Pluto-0013 | 25-29 | Male | Pre-vaccine, Post-1^st^ dose, Post-2^nd^ dose | Yes | Pfizer |
| Pluto-0014 | 35-39 | Female | Pre-vaccine, Post-1^st^ dose, Post-2^nd^ dose | Yes | Moderna |
| Pluto-0015 | 20-24 | Female | Pre-vaccine, Post-1^st^ dose, Post-2^nd^ dose | No | Pfizer |
| Pluto-0016 | 25-29 | Female | Pre-vaccine, Post-1^st^ dose, Post-2^nd^ dose | Yes | Pfizer |
| Pluto-0017 | 40-44 | Female | Pre-vaccine, Post-1^st^ dose, Post-2^nd^ dose | No | Moderna |
| Pluto-0018 | 40-44 | Female | Pre-vaccine, Post-1^st^ dose, Post-2^nd^ dose | No | Pfizer |
| Pluto-0019 | 30-34 | Female | Pre-vaccine, Post-1^st^ dose, Post-2^nd^ dose | No | Pfizer |
| Pluto-0020 | 35-39 | Male | Pre-vaccine, Post-1^st^ dose, Post-2^nd^ dose | Yes | Moderna |

**Supplementary Table 3: Characteristics of individuals shown in Figure 2 and S Figure 1.**

| **Participant ID** | **Age Bracket** | **Sex** | **Time points included in this study** | **SARS CoV-2 infection prior to vaccination** | **Vaccine Type** |
| --- | --- | --- | --- | --- | --- |
| OMI-001 | 30-39 | Female | Post-Vax, Post-Boost | No | Moderna |
| OMI-002 | 18-29 | Female | Post-Vax, Post-Boost | No | Moderna |
| OMI-003 | 40-49 | Male | Post-Vax, Post-Boost | No | Moderna |
| OMI-004 | 40-49 | Female | Post-Vax, Post-Boost | No | Moderna |
| OMI-005 | 40-49 | Female | Post-Vax, Post-Boost | No | Moderna |
| OMI-006 | 40-49 | Male | Post-Vax, Post-Boost | No | Moderna |
| OMI-007 | 50-59 | Female | Post-Vax, Post-Boost | No | Moderna |
| OMI-008 | 60-69 | Female | Post-Vax, Post-Boost | No | Moderna |
| OMI-009 | 30-39 | Female | Post-Vax, Post-Boost | No | Moderna |
| OMI-010 | 18-29 | Female | Post-Vax, Post-Boost | No | Moderna |
| OMI-011 | 40-49 | Female | Post-Vax, Post-Boost | No | Pfizer |
| OMI-012 | 18-29 | Female | Post-Vax, Post-Boost | No | Pfizer |
| OMI-013 | 40-49 | Female | Post-Vax, Post-Boost | No | Pfizer |
| OMI-014 | 50-59 | Female | Post-Vax | No | Pfizer |
| OMI-015 | 18-29 | Female | Post-Vax, Post-Boost | No | Pfizer |
| OMI-016 | 70-79 | Male | Post-Vax, Post-Boost | No | Pfizer |
| OMI-017 | 30-39 | Female | Post-Vax, Post-Boost | No | Pfizer |
| OMI-018 | 30-39 | Female | Post-Vax, Post-Boost | No | Pfizer |
| OMI-019 | 40-29 | Male | Post-Vax, Post-Boost | No | Pfizer |
| OMI-020 | 40-49 | Female | Post-Vax, Post-Boost | No | Pfizer |
| OMI-021 | 40-49 | Female | Post-Infection, Post-Vax | Yes | Pfizer |
| OMI-022 | 30-39 | Female | Post-Infection, Post-Vax | Yes | Pfizer |
| OMI-023 | 30-39 | Female | Post-Infection, Post-Vax | Yes | Pfizer |
| OMI-024 | 30-39 | Male | Post-Infection, Post-Vax | Yes | Pfizer |
| OMI-028 | 18-29 | Female | Post-Infection, Post-Vax | Yes | Moderna |
| OMI-029 | 30-39 | Female | Post-Infection, Post-Vax | Yes | Pfizer |
| OMI-032 | 50-59 | Male | Post-Infection, Post-Vax | Yes | Pfizer |
| OMI-027 | 40-49 | Female | Post-Infection, Post-Vax | Yes | Pfizer |
| OMI-033 | 30-39 | Female | Post-Infection, Post-Vax | Yes | Pfizer |
| OMI-034 | 40-49 | Male | Post-Infection, Post-Vax | Yes | Pfizer |
| OMI-035 | 30-39 | Female | Post-Infection, Post-Vax | Yes | Pfizer |
| OMI-025 | 50-59 | Male | Post-Infection | Yes | No vax |
| OMI-026 | 30-39 | Male | Post-Infection | Yes | No vax |
| OMI-030 | 40-49 | Female | Post-Infection | Yes | No vax |
| OMI-031 | 30-39 | Female | Post-Infection | Yes | No vax |
| OMI-036 | 50-59 | Male | Post-Vax | Yes | Moderna |
| OMI-037 | 30-39 | Male | Post-Vax | Yes | Moderna |
| OMI-038 | 18-29 | Male | Post-Vax | Yes | Moderna |
| OMI-039 | 30-39 | Female | Post-Vax | Yes | Moderna |
| OMI-040 | 60-69 | Female | Post-Vax | Yes | Moderna |
| OMI-041 | 40-49 | Female | Post-Vax | Yes | Moderna |
| OMI-042 | 40-49 | Female | Post-Vax | Yes | Moderna |
| OMI-043 | 60-69 | Male | Post-Vax | Yes | Moderna |
| OMI-044 | 40-49 | Male | Post-Vax | Yes | Moderna |
| OMI-045 | 30-39 | Male | Post-Boost | Yes | Pfizer |
| OMI-046 | 30-39 | Female | Post-Boost | Yes | Pfizer |
| OMI-048 | 60-69 | Male | Post-Boost | Yes | Pfizer |
| OMI-049 | 60-69 | Male | Post-Boost | Yes | Pfizer |
| OMI-050 | 30-39 | Female | Post-Boost | Yes | Pfizer |
| OMI-051 | 50-59 | Female | Post-Boost | Yes | Pfizer |
| OMI-052 | 50-59 | Female | Post-Boost | Yes | Pfizer |
| OMI-053 | 18-29 | Female | Post-Boost | Yes | Pfizer |
| OMI-054 | 30-39 | Female | Post-Boost | Yes | Pfizer |

**References**

1. Yeager CL, Ashmun RA, Williams RK, Cardellichio CB, Shapiro LH, Look AT, Holmes KV. 1992. Human aminopeptidase N is a receptor for human coronavirus 229E. Nature 357:420-2.

2. Hofmann H, Pyrc K, van der Hoek L, Geier M, Berkhout B, Pöhlmann S. 2005. Human coronavirus NL63 employs the severe acute respiratory syndrome coronavirus receptor for cellular entry. Proc Natl Acad Sci U S A 102:7988-93.

3. Letko M, Marzi A, Munster V. 2020. Functional assessment of cell entry and receptor usage for SARS-CoV-2 and other lineage B betacoronaviruses. Nat Microbiol 5:562-569.

4. Li W, Moore MJ, Vasilieva N, Sui J, Wong SK, Berne MA, Somasundaran M, Sullivan JL, Luzuriaga K, Greenough TC, Choe H, Farzan M. 2003. Angiotensin-converting enzyme 2 is a functional receptor for the SARS coronavirus. Nature 426:450-4.

5. Wells HL, Letko M, Lasso G, Ssebide B, Nziza J, Byarugaba DK, Navarrete-Macias I, Liang E, Cranfield M, Han BA, Tingley MW, Diuk-Wasser M, Goldstein T, Johnson CK, Mazet JAK, Chandran K, Munster VJ, Gilardi K, Anthony SJ. 2021. The evolutionary history of ACE2 usage within the coronavirus subgenus. Virus Evol 7:veab007.

6. Raj VS, Smits SL, Provacia LB, van den Brand JM, Wiersma L, Ouwendijk WJ, Bestebroer TM, Spronken MI, van Amerongen G, Rottier PJ, Fouchier RA, Bosch BJ, Osterhaus AD, Haagmans BL. 2014. Adenosine deaminase acts as a natural antagonist for dipeptidyl peptidase 4-mediated entry of the Middle East respiratory syndrome coronavirus. J Virol 88:1834-8.

7. Woo PC, Lau SK, Li KS, Tsang AK, Yuen KY. 2012. Genetic relatedness of the novel human group C betacoronavirus to Tylonycteris bat coronavirus HKU4 and Pipistrellus bat coronavirus HKU5. Emerg Microbes Infect 1:e35.

8. Yang Y, Du L, Liu C, Wang L, Ma C, Tang J, Baric RS, Jiang S, Li F. 2014. Receptor usage and cell entry of bat coronavirus HKU4 provide insight into bat-to-human transmission of MERS coronavirus. Proc Natl Acad Sci U S A 111:12516-21.

9. Woo PC, Wang M, Lau SK, Xu H, Poon RW, Guo R, Wong BH, Gao K, Tsoi HW, Huang Y, Li KS, Lam CS, Chan KH, Zheng BJ, Yuen KY. 2007. Comparative analysis of twelve genomes of three novel group 2c and group 2d coronaviruses reveals unique group and subgroup features. J Virol 81:1574-85.

10. Corman VM, Ithete NL, Richards LR, Schoeman MC, Preiser W, Drosten C, Drexler JF. 2014. Rooting the phylogenetic tree of middle East respiratory syndrome coronavirus by characterization of a conspecific virus from an African bat. J Virol 88:11297-303.

11. Xiong Q, Cao L, Ma C, Tortorici MA, Liu C, Si J, Liu P, Gu M, Walls AC, Wang C, Shi L, Tong F, Huang M, Li J, Zhao C, Shen C, Chen Y, Zhao H, Lan K, Corti D, Veesler D, Wang X, Yan H. 2022. Close relatives of MERS-CoV in bats use ACE2 as their functional receptors. Nature 612:748-757.

12. Huang C, Liu WJ, Xu W, Jin T, Zhao Y, Song J, Shi Y, Ji W, Jia H, Zhou Y, Wen H, Zhao H, Liu H, Li H, Wang Q, Wu Y, Wang L, Liu D, Liu G, Yu H, Holmes EC, Lu L, Gao GF. 2016. A Bat-Derived Putative Cross-Family Recombinant Coronavirus with a Reovirus Gene. PLoS Pathog 12:e1005883.

13. Wu Z, Yang L, Ren X, Zhang J, Yang F, Zhang S, Jin Q. 2016. ORF8-Related Genetic Evidence for Chinese Horseshoe Bats as the Source of Human Severe Acute Respiratory Syndrome Coronavirus. J Infect Dis 213:579-83.

14. Schultze B, Herrler G. 1994. Recognition of cellular receptors by bovine coronavirus. Arch Virol Suppl 9:451-9.

15. Hulswit RJG, Lang Y, Bakkers MJG, Li W, Li Z, Schouten A, Ophorst B, van Kuppeveld FJM, Boons GJ, Bosch BJ, Huizinga EG, de Groot RJ. 2019. Human coronaviruses OC43 and HKU1 bind to 9-. Proc Natl Acad Sci U S A 116:2681-2690.

16. Yuan Y, Zu S, Zhang Y, Zhao F, Jin X, Hu H. 2021. Porcine Deltacoronavirus Utilizes Sialic Acid as an Attachment Receptor and Trypsin Can Influence the Binding Activity. Viruses 13.

17. Wang L, Maddox C, Terio K, Lanka S, Fredrickson R, Novick B, Parry C, McClain A, Ross K. 2020. Detection and Characterization of New Coronavirus in Bottlenose Dolphin, United States, 2019. Emerg Infect Dis 26:1610-1612.
